# 96.5 Using Computer Vision-Based Algorithms Trained on Mobile-Device Camera Images for Monitoring Burn Wound Healing

**DOI:** 10.1093/jbcr/irac012.099

**Published:** 2022-03-23

**Authors:** Hannah O Chan, Rakesh Joshi, Alexander Morzycki, Andrew C Pun, Joshua N Wong, Collins Hong

**Affiliations:** Skinopathy, Inc.; Data Scientist, Skinopathy Inc.; Division of Plastic and Reconstructive Surgery, University of Alberta, Edmonton, Canada; Data Science Intern, Skinopathy Inc.; Division of Plastic Surgery, Department of Surgery, University of Alberta

## Abstract

**Introduction:**

The appropriate characterization of burn depth and healing is paramount. Unfortunately, the accuracy of approximating thermal injury depth among all physicians is poor. While tools to improve detection accuracy, including laser doppler imaging and laser speckle imaging exist, these technologies are expensive and limited to specialized burn referral centres. They also do not provide an easy means for quantitative, interval tracking of burn healing. Considering these limitations, the application of artificial intelligence has garnered significant interest. We herein present the use of three novel machine learning and computer vision-based algorithms to track burn wound healing.

**Methods:**

Convolutional neural network (CNN) models, were trained on 1800 2D color burn images, to classify them into four burn severities. These CNNs were used to develop saliency algorithms that identify the highest “attention” pixels used to recognize burns. Image-based algorithms that count these attention pixels of the CNN, count pixels representing red granulation of burns, and measure burns, were also developed. As proof-of-concept, we tracked the healing of a localized burn on a 25-year-old female patient. The patient suffered a scald on the dorsum of the foot, resulting in a deep partial-thickness burn. Opting out of surgical intervention, the patient visited the hospital over a 6-week period for treatment with non-adhesive dressings and silver nitrate. High-resolution images of the burn, with and without a fiducial marker, were captured with a smartphone camera every 7-days. Images were taken under institutional lighting and used as algorithmic inputs.

**Results:**

Data analyses indicate that the healing of the open-wound area was accurately measured in millimetres (+/- 1.7 mm error) using a fiducial marker (18.3 mm diameter). The open-wound area shrank consistently from week 1 to week 6 seen in (Figure 1. a-b). The normalized, 2D colour images, where the “red” pixel value was counted (Figure 1. a-b), confirms the reduction of the red granulation in the wound. The saliency algorithm also measured a percentage reduction in the machine learning model’s total attention pixels over the 6-week period (Figure 1. c-d). This suggests that the model was less discerning of the healing burn wound over time, suggesting burn healing, which was also clinically validated.

